# Socioeconomic inequalities in health behaviors: exploring mediation pathways through material conditions and time orientation

**DOI:** 10.1186/s12939-021-01522-2

**Published:** 2021-08-14

**Authors:** Andrea L. Mudd, Frank J. van Lenthe, Sanne E. Verra, Michèlle Bal, Carlijn B. M. Kamphuis

**Affiliations:** 1 Department of Interdisciplinary Social Science- Social Policy and Public Health, Utrecht University, PO Box 80140, 3508 TC Utrecht, The Netherlands; 2 Department of Public Health, Erasmus University Medical Centre, PO Box 2040, 3000 CA Rotterdam, The Netherlands; 3 Department of Human Geography and Spatial Planning, Utrecht University, PO Box 80140, 3508 TC Utrecht, The Netherlands

**Keywords:** Socioeconomic inequalities, Health behavior, Material conditions, Time orientation, Sequential mediation, Structural equation modeling, The Netherlands

## Abstract

**Background:**

Socioeconomic inequalities in health behaviors have been attributed to both structural and individual factors, but untangling the complex, dynamic pathways through which these factors influence inequalities requires more empirical research. This study examined whether and how two factors, material conditions and time orientation, sequentially impact socioeconomic inequalities in health behaviors.

**Methods:**

Dutch adults 25 and older self-reported highest attained educational level, a measure of socioeconomic position (SEP); material conditions (financial strain, housing tenure, income); time orientation; health behaviors including smoking and sports participation; and health behavior-related outcomes including body mass index (BMI) and self-assessed health in three surveys (2004, 2011, 2014) of the longitudinal GLOBE (Dutch acronym for “Health and Living Conditions of the Population of Eindhoven and surroundings”) study. Two hypothesized pathways were investigated during a ten-year time period using sequential mediation analysis, an approach that enabled correct temporal ordering and control for confounders such as baseline health behavior.

**Results:**

Educational level was negatively associated with BMI, positively associated with sports participation and self-assessed health, and not associated with smoking in the mediation models. For smoking, sports participation, and self-assessed health, a pathway from educational level to the outcome mediated by time orientation followed by material conditions was observed.

**Conclusions:**

Time orientation followed by material conditions may play a role in determining socioeconomic inequalities in certain health behavior-related outcomes, providing empirical support for the interplay between structural and individual factors in socioeconomic inequalities in health behavior. Smoking may be determined by prior smoking behavior regardless of SEP, potentially due to its addictive nature. While intervening on time orientation in adulthood may be challenging, the results from this study suggest that policy interventions targeted at material conditions may be more effective in reducing socioeconomic inequalities in certain health behaviors when they account for time orientation.

**Supplementary Information:**

The online version contains supplementary material available at 10.1186/s12939-021-01522-2.

## Background

There is evidence of a socioeconomic gradient in health behaviors in the Netherlands, meaning that those with incrementally lower socioeconomic position (SEP) engage in fewer health-promoting behaviors [1–4]. Health behaviors have been shown to explain an important part of the Dutch socioeconomic gradient in mortality and other health outcomes [5, 6]. Understanding the concrete pathways and mechanisms linking SEP and health behaviors is therefore crucial for designing effective policy interventions aimed at reducing socioeconomic inequalities in health [7].

Untangling these mechanisms is a complex task, as socioeconomic inequalities in health behaviors are increasingly understood to be driven by dynamic, interlinked mechanisms [7–9], including the interplay between structural and individual factors [10, 11]. One structural factor shown to influence socioeconomic inequalities in health behaviors is material conditions [12–15]. Material conditions, or consumption possibilities such as income and assets, can be considered a tangible reflection of the material environment and could be a barrier to developing or maintaining a healthy lifestyle. Many health-promoting behaviors require a financial investment, such as a gym membership. Time orientation, a measure of perceived time that refers to a person’s cognitive involvement in the past, present, or future [16], is an individual factor that has been found to impact socioeconomic inequalities in health behavior [17–19]. Individuals’ attitudes about time may affect how they invest the resources at their disposal. Specifically, time orientation may impact the decision to engage in health-promoting behaviors or abstain from behaviors harmful to health in the present that only produce health benefits in the future.

Time orientation and material conditions have so far been considered to influence health behavior through isolated pathways, as cited above, but it is likely that the two factors are sequentially interlinked. Two potential sequential pathways through which time orientation and material conditions impact socioeconomic inequalities in health behaviors in the Netherlands are investigated in this study, with educational level used as a measure of SEP.

The first hypothesized pathway is grounded in the scarcity theory. According to the scarcity theory, dealing with scarce resources hinders individuals’ cognitive capacity to pay attention, make good decisions, stick with plans, and resist temptations [20]. Individuals from lower socioeconomic backgrounds may have poorer or less stable material conditions. The cognitive burden of dealing with unfavorable material conditions may result in the experience of scarcity, which could hinder the ability to focus on the future, impacting time orientation [21, 22]. In turn, a more present-focused time orientation could limit the uptake of health-promoting behaviors because while the future effects of these behaviors are uncertain, their costs are incurred in the present. The first hypothesized pathway through which time orientation and material conditions impact socioeconomic inequalities in health behavior is:

- Hypothesis 1: *SEP→Material conditions→Time orientation→Health behavior*

In the second hypothesized pathway, time orientation precedes material conditions. Having a lower SEP could shape a more present-focused time orientation, which could negatively affect individuals’ abilities to achieve favorable material conditions and, in turn, lead to less healthy behavior. Previous studies have shown that those with a lower SEP may be less likely to have a future-focused time orientation [23], which could be due to a variety of reasons. Experiencing a lack of status and social or political influence could lead to a low perceived ability to influence the future [24]. Processes in childhood, such as socialization by parents [25], or exposure to poverty in childhood, which could lead to the belief that the future is uncertain and difficult to plan for [26], could also explain why time orientation is influenced by SEP. In turn, individuals with a present time orientation may be less likely to save money [27], potentially leading to more financial strain or the inability to buy a home. Less favorable material conditions could then hinder one’s ability to develop and maintain health-promoting behaviors. Unlike the first hypothesized pathway, which posits that time orientation could vary in the relatively short term in response to changes in material conditions, the second pathway considers time orientation to be shaped by SEP over a longer period of time. The second hypothesized pathway through which time orientation and material conditions impact socioeconomic inequalities in health behavior is:

- Hypothesis 2: *SEP→Time orientation→Material conditions→Health behavior*

The overall aim of this study is to understand whether and in what order time orientation and material conditions influence socioeconomic inequalities in health behaviors among adults. Understanding if and how this sequential relationship exists could help policymakers move beyond interventions that target single factors in isolation towards interventions that account for how multiple factors, at structural and individual levels, are interlinked in their influence on socioeconomic inequalities in health behavior. Another contribution this study makes to the existing literature on socioeconomic inequalities is the use of sequential mediation analysis and longitudinal data to test the hypothesized pathways. The mediation models will account for confounding by baseline levels of health behavior, which has seldom been done in studies on this topic. Most existing studies have focused on the size of the total contribution of certain groups of factors to the explanation of inequalities in health behavior over the life course (e.g., [1, 28]). From an intervention-focused perspective, it is important to account for baseline differences in health behaviors between socioeconomic groups of adults; the relationships between the factors influencing health behaviors and health behaviors themselves are likely confounded by prior levels of health behavior. Besides impacting health behavior over time, prior unhealthy behavior could lead to health complaints, which may result in a more present-focused time orientation, and poorer material conditions due to, for instance, job loss. Controlling for prior health behavior will enable a clearer understanding of the causal pathways outlined in the two hypotheses, which is important for assessing whether intervening on time orientation and material conditions could help reduce socioeconomic inequalities in health behavior in adulthood.

## Methods

### The GLOBE data

GLOBE (Dutch acronym for “Health and Living Conditions of the Population of Eindhoven and surroundings”) is a prospective cohort study focused on understanding socioeconomic inequalities in a representative sample of the population in the Eindhoven area of the Netherlands. Data from 18,973 respondents were collected via an initial postal survey in 1991 [29], and two subsamples of these baseline participants were selected to form the longitudinal GLOBE cohort (*N* = 5667). The longitudinal cohort was followed up with postal surveys in 1997, 2004, 2011, and 2014, and two new samples consisting of adults residing in the Eindhoven area were added to the study in 2004 and 2014 to compensate for attrition. Data from respondents who participated in the 2004, 2011, and 2014 surveys who were at least 25 years old in 2004 (*N* = 2692) were used for the analyses described in this paper. These three waves of data were chosen because they included variables required to test the two hypotheses.

Details about the study design and sampling methods are described in Additional file 1, and more in-depth discussions of the GLOBE sample are published elsewhere [29, 30]. The use of personal data in the GLOBE study is in compliance with the Dutch Personal Data Protection Act and the Municipal Database Act; the study is registered with the Dutch Data Protection Authority (number 1248943).

### Measures used in analyses

#### Educational level, a measure of SEP

The highest attained educational level at baseline (2004) was used as a measure of SEP. Traditionally, educational level has been the most important measure of social stratification in Dutch society [31], so while it does not fully represent SEP, educational level is considered a valid measure of SEP to use in this study. Respondents aged 25 years and older at baseline were included in the study sample because they were assumed to have completed their education. Four education categories were defined according to the International Standard Classification of Education (ISCED): high (higher professional education and university; ISCED 5–7), middle (intermediate professional and higher general education; ISCED 3–4), low (lower professional and intermediate general education; ISCED 2), and lowest (no or primary education; ISCED 0–1).

#### Material conditions: financial strain, housing tenure, income

Data on financial strain, housing tenure, and income from 2011 were used as three separate measures of material conditions in the analyses. These commonly used measures were investigated separately in order to identify whether the measures differed in their importance for the pathways investigated in this study. Financial strain was recorded by asking respondents whether they experienced any difficulties paying for food, rent, electricity, and so forth during the past year; possible answers were "no difficulty at all", "some difficulty", and "great difficulty". For housing tenure, respondents indicated whether they owned or rented their home. Income is sometimes used as a measure of SEP, but it has also been used as an indicator of material conditions in studies that measure SEP with educational level [32, 33]. In this study, income is considered a useful representation of individuals’ financial resources and was measured by asking respondents to select the range their monthly income falls into.

#### Time orientation

In 2011, time orientation was measured by asking participants to what extent they agreed with each of a set of ten statements about the present and the future on a five-level scale ranging from “strongly disagree” to “strongly agree” (Chronbach’s alpha = 0.60). The statements, such as “I often think about how my actions today will affect my health when I am older” and “What happens to me in the future is out of my control” are from a brief scale developed to measure time orientation in African American women [34]. A mean time orientation score was calculated by averaging the responses to the individual questions, accounting for whether each statement was positively or negatively phrased. Possible time orientation scores ranged from 1 to 5, with lower scores indicating a preference for the present and higher scores indicating a preference for the future; the resulting scores ranged from 1.1 to 4.8. Though it is just one of many existing measures used to study the perception of time [16], the brief scale used in this study is a valid measure of time orientation as it is defined in this study.

#### Health behavior-related outcomes

Smoking behavior and sports participation in 2004 and 2014 were measures of health behavior included in the analyses. BMI and self-assessed health in 2004 and 2014 were included as outcomes related to health behavior; BMI represents a combined effect of physical activity and dietary intake, and self-assessed health can be considered a general representation of a healthy lifestyle.

Smoking behavior was self-reported based on the question “Do you smoke?” and a series of questions about the frequency and type of tobacco product(s) consumed; answers were dichotomized into non-smoker and current smoker. Sports participation was collected using the validated Short QUestionnaire to ASsess Health-enhancing physical activity (SQUASH) [35]. Respondents were asked to report the average frequency and duration of their sports activities per week. In line with a recent study using GLOBE data [5], participants were categorized as active (> 2 h/week), moderately active (1–2 h/week), little active (< 1 h/week), or inactive (0 h/week). Since very few respondents belonged to the little active category (1.8%), this group was combined with the moderately active category for the analyses. Self-reported weight and height were used to calculate BMI (weight/height^2^), which was then classified as underweight (BMI ≤ 18.5), normal (BMI > 18.5–25), overweight (BMI > 25–30), and obese (BMI > 30). The underweight and normal BMI categories were combined in the analyses due to few respondents reporting an underweight BMI (0.9%). Self-assessed health was measured by asking respondents to complete the sentence, “In general, would you say your health is …” with options on a five-level scale ranging from “poor” to “excellent”.

#### Baseline demographic covariates

Age in years and gender were collected in 2004. For gender, respondents indicated whether they were male or female. A binary variable indicating whether a respondent was female or not was used in the analyses. Additional file 2 contains a detailed overview of all variables included in the analyses.

### Sequential mediation analyses

The pathways through which time orientation and material conditions impact socioeconomic inequalities in health behaviors were tested using sequential mediation analysis. In a sequential mediation analysis, the mediators (here, time orientation and material conditions) are posited to at least partially explain the causal relationship between the exposure (educational level) and the (health behavior-related) outcome in a specific order. The hypothesized pathways were investigated using structural equation models (SEMs), which are systems of linked regression-style equations that estimate complex, dynamic relationships in a set of variables. The SEM approach was chosen because of the ability to test complicated hypotheses, handle ordinal dependent variables in a straightforward way, and calculate model fit statistics [36]. For every outcome, separate SEMs were estimated for each of the three measures of material conditions using a weighted least squares (WLS) estimator, the standard method applied for SEMs with ordered dependent variables [37].

Each SEM was comprised of three simultaneously fitted equations. Constant terms and coefficients for the baseline covariates (in Eqs. 1 and 2, age and gender and in Eq. 3, age, gender, and baseline health behavior-related outcome) are not shown.

1 $$ Mediator\ 1\sim \kern0.5em a1\times Exposure+ Baseline\ covariates+\varepsilon 1 $$

2 $$ Mediator\ 2\sim a2\times Exposure+d\times Mediator\ 1+ Baseline\ covariates+\varepsilon 2 $$

3 $$ Outcome\sim c^{\prime}\times Exposure+b1\times Mediator\ 1+b2\times Mediator\ 2+ Baseline\ covariates+\varepsilon 3 $$

**Fig. 1:**
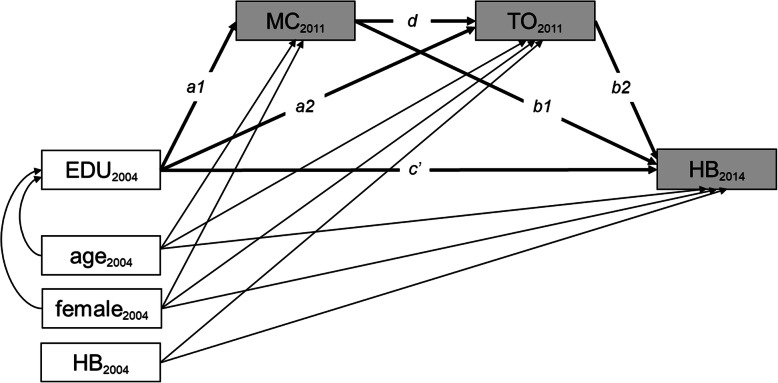
Path diagram (Hypothesis 1). EDU: educational level, HB: health behavior-related outcome, MC: a measure of material conditions, TO: time orientation. Indirect effect through material conditions followed by time orientation= a1*d*b2, direct effect = c’. Shaded boxes indicate variables that are dependent in at least one equation, and unshaded boxes represent independent variables

**Fig. 2:**
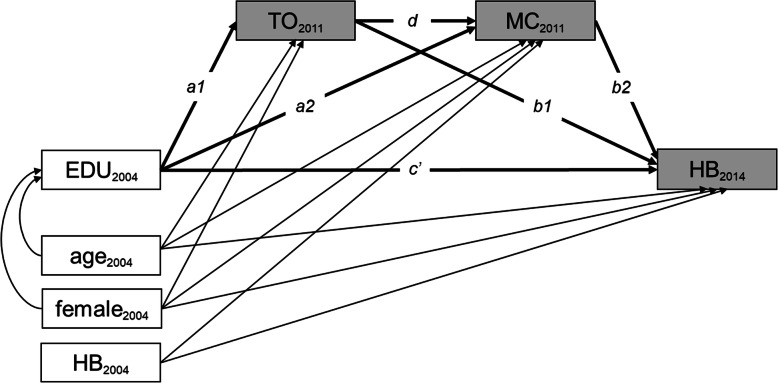
Path diagram (Hypothesis 2). EDU: educational level, HB: health behavior-related outcome, MC: a measure of material conditions, TO: time orientation. Indirect effect through time orientation followed by material conditions = a1*d*b2, direct effect = c’. Shaded boxes indicate variables that are dependent in at least one equation, and unshaded boxes represent independent variables

For each hypothesis, *Mediator 1* and *Mediator 2* are defined according to the order of the factors (time orientation and material conditions) tested in each hypothesis. The direct effect of the exposure on the outcome, controlling for the mediators, is *c’*. The indirect effect of the exposure on the outcome through the sequence of mediators is a1*d*b2; when interpreting the indirect effect, the effect size and statistical significance of the pathway in its entirety is of interest, not the effect size and statistical significance of each component of the pathway [38]. The total indirect effect of the exposure on the outcome through the mediators, consisting of the indirect effect through the sequence of mediators along with the indirect effects of each mediator individually, is the sum of a1*d*b2, a1*b1, and a2*b2 [39]. The error terms (ɛ1, ɛ2, ɛ3) were assumed to be uncorrelated and multivariate normally distributed, assumptions required for the definition of indirect and direct effects [40]. A statistically significant total effect, which is the sum of the direct and total indirect effects, is not a prerequisite for investigating indirect effects when the analysis is based on mediation hypotheses [41]. This study is based on two hypotheses, grounded in theory, about the presence of sequential mediation pathways, so total effects are not reported and, instead, the direct and indirect effects through the sequences of mediators are the focus of the analyses presented in this text. Given this focus on examining specific pathways, indirect effects through each mediator individually and total indirect effects were estimated but are not discussed in detail. For completeness, details on the indirect effects of each mediator individually, total indirect effects, and total effects are provided in Additional file 3. In this article, further mentions of “indirect effects” refer to indirect effects through sequences of two mediators. The path diagrams in Figs. 1 and 2 display the relationships estimated to test Hypotheses 1 and 2, respectively.

Statistically significant direct effects indicate pathways from educational level to health behavior that do not run through the mediators, and statistically significant indirect effects indicate pathways that help explain educational inequalities in health behavior. Given this study’s focus on identifying meaningful pathways, the interpretation of results will be focused on which indirect effects are statistically significant, referred to as the null hypothesis perspective [41]. Another approach is the effect size perspective, in which the percentage of the relationship between the exposure and outcome explained by a sequence of mediators is calculated by dividing the total effect by the indirect effect through that sequence of mediators.

Several assumptions required for causal interpretation of indirect and direct effects from sequential mediation analyses were addressed in the study design [42]. Three waves of GLOBE data were used to establish a temporal sequence between the exposure (2004), the mediators (2011), and the outcome (2014), preventing the possibility of observed relationships being due to reverse causality. Age and gender at baseline (2004) were included as covariates in all three SEM equations because they are likely important confounders of the relationships between the exposure, the mediators, and the outcomes. Health behavior at baseline (2004) was controlled for in the equation predicting health behavior in 2014 to account for mediator-outcome confounding [43, 44]. An implication of this study design is that the analysis estimates changes in health behavior-related outcomes through the hypothesized pathways during a period of 10 years (2004–2014). This design suits the study’s aim to understand whether intervening on time orientation and material conditions in adulthood could lead to a larger improvement in health behaviors among those with lower education than among those with higher education, or, put otherwise, a reduction in educational inequalities in health behaviors.

Using multiple imputation, 20 imputed datasets were generated to account for the bias introduced by missing data. Because certain responses would hinder the interpretability of the results, those who reported having an “Other” educational level (*N* = 8) and those who reported an “Unknown/ refuse to say” income (*N* = 254) in the imputed datasets were excluded from the mediation analyses. The sequential mediation SEMs were estimated on each of the imputed datasets, then parameter estimates and standard errors were pooled using Rubin’s rule [45, 46]. Details about the handling of missing data can be found in Additional file 4.

All analyses were performed in R (version 3.6.0). The lavaan (version 0.6-6) and semTools (version 0.5-3) packages were used for mediation analyses and the mice package (version 3.9.0) was used for multiple imputation.

## Results

### Descriptive statistics of GLOBE respondents

Descriptive statistics from the non-imputed data for the full sample and by educational level are shown in Table 1. The majority of respondents had high (33%), middle (22%), or low (33%) education. Only 7% had the lowest level of education, and educational level was missing for 4% of respondents. In the full sample, the average age was 55.2 years and there were slightly more females than males. Educational gradients were observed for time orientation, all measures of material conditions, and all health behavior-related outcomes.

**Table 1:** Descriptive statistics of GLOBE respondents

		Educational level (2004)			
	Total	Lowest	Low	Middle	High
	N = 2692^a^	*N* = 196	*N* = 896	*N* = 604	*N* = 891
**Age (2004)**					
Mean (SD)	55.2 (13.6)	63.1 (10.9)	59.9 (10.8)	50.9 (13.5)	50.5 (13.9)
**Gender (2004), %**					
Female	53.6	65.8	64.1	47.8	42.9
Male	46.4	34.2	35.9	52.2	57.0
NA	0.0	0.0	0.0	0.0	0.1
**Time orientation (2011)**					
Mean (SD)	3.2 (0.5)	3.0 (0.5)	3.2 (0.5)	3.3 (0.5)	3.4 (0.4)
Range	1.1–4.8	1.2–4.2	1.1–4.7	1.8–4.8	1.8–4.5
NA, %	20.2	19.9	16.6	20.5	23.5
**Financial strain (2011), %**					
No difficulty	76.7	57.7	72.8	74.5	87.0
Some difficulty	19.2	32.7	22.5	21.7	10.7
Great difficulty	2.9	7.1	2.9	3.6	1.7
NA	1.2	2.6	1.8	0.2	0.7
**Housing tenure (2011), %**					
Owned home	68.6	26.0	57.7	72.8	87.8
Rented home	30.2	72.4	40.4	26.2	12.0
NA	1.2	1.5	1.9	1.0	0.2
**Monthly income group (2011), %**					
0–1200 Euros	7.7	28.1	10.7	4.0	1.7
1200–1800 Euros	17.3	33.2	25.8	16.1	5.6
1800–2600 Euros	25.4	13.8	30.1	30.8	18.9
2600–4000 Euros	26.5	4.6	15.6	32.3	39.7
> 4000 Euros	11.7	0.5	3.3	8.6	25.8
Unknown/refuse to say/NA	11.4	19.9	14.4	8.3	8.3
**Smoking status (2014), %**					
Non-smoker	87.6	87.6	81.6	86.4	85.3
Smoker	11.3	15.8	11.6	14.2	8.1
NA	1.2	2.6	2.0	0.5	0.3
**Sports participation (2014), %**					
Inactive	40.3	62.2	46.9	36.9	30.3
Little active	1.8	1.5	1.3	1.3	2.9
Moderately active	12.9	5.1	11.0	15.1	15.8
Active	34.9	17.3	29.0	37.6	43.5
NA	10.1	13.8	11.7	9.1	7.4
**BMI (2014), %**					
Underweight	0.9	1.5	1.0	0.5	0.9
Normal	41.3	36.7	33.9	39.1	51.3
Overweight	41.0	34.7	45.5	44.4	35.9
Obese	14.5	22.4	17.0	14.4	10.3
NA	2.3	4.6	2.6	1.7	1.6
**Self-assessed health (2014), %**					
Poor	2.2	7.1	2.6	1.7	1.0
Fair	16.5	33.2	22.8	11.4	8.3
Good	50.7	47.4	54.0	51.5	48.1
Very good	22.1	10.2	15.0	26.5	29.4
Excellent	7.9	0.5	4.8	8.4	12.8
NA	0.7	1.5	0.9	0.5	0.3

*P*-values from Pearson chi-square tests for independence for all variables by educational level were < 0.001, indicating that the null hypotheses of each variable being independent of educational level could be rejected

*BMI* body mass index, *NA* missing values, *SD* standard deviation

^a^*N* = 105 respondents had missing data for educational level (*N* = 97) or reported having “Other” education (*N* = 8)

### Results of the sequential mediation analyses

The effect sizes and statistical significance levels of direct and indirect effects for every model estimated to test Hypotheses 1 and 2 are listed in Tables 2 and 3, respectively. The fit statistics for the majority of the models were well within acceptable ranges (see Additional file 3 for details about model fit).

**Table 2:** Mediation results, Hypothesis 1: Educational level → Material conditions → Time orientation → Health behavior

Outcomes (in separate models)		Measure of material conditions (tested in separate models)					
		Financial strain → Time orientation		Housing tenure → Time orientation		Income group → Time orientation	
	*N*	IE	DE	IE	DE	IE	DE
*Health behaviors*							
**Smoking**	*2661*	−0.001	−0.010	0.001	0.009	0.003	0.009
**Sports participation**	*2420*	−0.001	0.064**	0.001	0.062*	0.002	0.043
*Health behavior-related outcomes*							
**BMI**	*2630*	0.000	−0.058*	0.000	−0.063**	0.000	−0.046
**Self-assessed health**	*2674*	0.001	0.145***	−0.001	0.132***	−0.002	0.095***

Results are shown for each of 12 separate models testing the effects of educational level on each of the four outcomes (smoking, sports participation, BMI, self-assessed health) through each of the three measures of material conditions (financial strain, housing tenure, income) followed by time orientation. In the models, all of which include the mediators, direct effects refer to the effect of educational level on health behavior that is not through the sequence of mediators and indirect effects refer to the effect of educational level on health behavior through the sequence of mediators

*BMI* body mass index, *DE* direct effect, *IE* indirect effect through material conditions followed by time orientation

Reported effects are statistically significant at *α = 0.1, **α = 0.05, ***α = 0.01

**Table 3:** Mediation results, Hypothesis 2: Educational level → Time orientation → Material conditions → Health behavior

Outcomes (in separate models)		Measure of material conditions (tested in separate models)					
		Time orientation → Financial strain		Time orientation → Housing tenure		Time orientation → Income group	
	*N*	IE	DE	IE	DE	IE	DE
*Health behaviors*							
**Smoking**	*2661*	0.002	−0.010	−0.001	0.009	−0.002**	0.009
**Sports participation**	*2420*	−0.002*	0.064**	0.001	0.062*	0.001*	0.043
*Health behavior-related outcomes*							
**BMI**	*2630*	0.000	−0.059*	0.000	−0.063**	0.000	−0.046
**Self-assessed health**	*2674*	−0.001*	0.146***	0.001	0.123***	0.002**	0.095***

Results are shown for each of 12 separate models testing the effects of educational level on each of the four outcomes (smoking, sports participation, BMI, self-assessed health) through time orientation followed by each of the three measures of material conditions (financial strain, housing tenure, income). In the models, all of which include the mediators, direct effects refer to the effect of educational level on health behavior that is not through the sequence of mediators and indirect effects refer to the effect of educational level on health behavior through the sequence of mediators

*BMI* body mass index, *DE* direct effect, *IE* indirect effect through time orientation followed by material conditions

Reported effects are statistically significant at *α = 0.1, **α = 0.05, ***α = 0.01

The direct effect estimates were the same for the models testing Hypotheses 1 and 2. The direct effect of educational level on smoking was not statistically significant regardless of the measure of material conditions included in the models. The direct effects of educational level on sports participation (for financial strain and housing), BMI (for financial strain and housing), and self-assessed health (for all measures of material conditions) were statistically significant. For these outcomes, the directions of the observed effects were as expected: a higher educational level led to a higher likelihood of spending more time on sports, a lower likelihood of having a high BMI, and a higher likelihood of reporting better self-assessed health. The largest direct effect sizes were observed for BMI and self-assessed health.

Several indirect effects in the sequential mediation models testing Hypothesis 2, that educational level influences health behavior through time orientation followed by material conditions, were statistically significant. Significant indirect effects of educational level on sports participation and self-assessed health through time orientation followed by either financial strain or income were observed, and a significant indirect effect of educational level on smoking through time orientation followed by income was observed. No significant indirect effects were found in the models with housing tenure as the measure of material conditions, and none of the indirect effects in the models testing Hypothesis 1 were significant. The indirect effect sizes ranged from − 0.002 to 0.003 in all models, meaning that the pathway through the sequences of mediators explained a small part of the total effect of educational level on health behavior. For example, the pathway through time orientation followed by income (indirect effect = 0.001) explained 0.9% of the relationship between educational level and self-assessed health (total effect = 0.112, see Additional file 3). When the individual mediating effects of time orientation and income are considered in addition to the sequential mediating effect, these factors explain 61.6% of the relationship between educational level and self-assessed health (total indirect effect = 0.069, see Additional file 3).

## Discussion

### Summary of main findings

When accounting for the mediators and baseline health behavior, educational level was directly associated with BMI, sports participation, and self-assessed health 10 years later but not with smoking. The second hypothesized pathway was supported by the mediation analyses, as time orientation followed by financial strain or income accounted for part of the influence of educational level on smoking, sports participation, and self-assessed health. No support was found for the first hypothesized pathway from educational level to health behavior through material conditions followed by time orientation.

### Interpretation of main findings

The estimates from this study indicate that educational level may have a larger direct effect on changes in behavior-related health outcomes (BMI and self-assessed health) than on changes in specific health behaviors (smoking and sports participation) over a 10-year period during adulthood. Because of its addictive nature, smoking may be relatively stable over time compared to other health behaviors, which could explain the insignificant direct effect of educational level on changes in smoking behavior over a 10-year period during adulthood.

These analyses did not support a pathway from educational level to health behavior through material conditions and time orientation, in that order (Hypothesis 1). Time orientation is often considered to remain stable over time, and the few empirical studies that examined changes in time orientation found evidence that it is influenced by educational level [47, 48]. This suggests that time orientation may be shaped by education over a longer period of time but may be less prone to short-term fluctuations in response to, for instance, material conditions. This could explain why we found evidence for a pathway from educational level to health behavior through time orientation followed by material conditions (Hypothesis 2) but not through material conditions followed by time orientation (Hypothesis 1).

Regarding Hypothesis 2, indirect effects were found for sports participation and self-assessed health when financial strain or income was included as a measure of material conditions and for smoking when income was included as a measure of material conditions. This pathway may have been observed for sports participation and self-assessed health, in particular, because a future time orientation and favorable material conditions might make engaging in more sports easier and more enjoyable. For smoking, income may play an especially important role as the means through which individuals can actually make decisions in line with their time orientation. Our findings suggest that for smoking, income may play a more important role than educational level, since the direct effects of educational level on smoking were insignificant in all models. This finding is not surprising as the strong relationship between income and smoking behavior is well-documented [49]. Young adulthood has been shown to be an important period of changes in BMI [50]. Since this study examined a 10-year period of adulthood above the age of 25, it is possible that important changes in BMI earlier in life were not captured. The sequence of time orientation followed by housing tenure did not mediate the relationship between educational level and any of the outcomes. This suggests that housing tenure does not play as important a role in the pathway as financial strain and income, perhaps because it is less variable over time than income and financial strain.

Though small in size, the indirect effects observed in the models testing the pathway from educational level to health behavior through time orientation followed by material conditions are noteworthy. The indirect effect sizes represent the contribution of the particular pathway, time orientation followed by material conditions, to the relationship between educational level and health behavior. They make up only one part of the total indirect effect, which also accounts for the individual contributions of each mediator to the relationship between educational level and health behavior (see Additional file 3) [51]. The pathway-specific indirect effects indicate the importance of a particular process in explaining educational inequalities in health behavior, which is why their statistical significance is meaningful despite small effect sizes [41]. The similarity in effect sizes between the models testing each hypothesis was expected, as these indirect effects represent the same part of the pathway regardless of the order they are modelled in. To summarize, the statistically significant indirect effects shed light on the processes determining socioeconomic inequalities in a set of health behaviors. Specifically, the effects were consistent within outcomes but differed between certain outcomes, highlighting that the processes underlying socioeconomic inequalities in smoking, sports participation, and self-assessed health may differ from those for BMI.

### Methodological considerations

This study focused on complex interplays using sequential mediation analysis. This method, which is not yet commonly used in public health research, provided novel insight into the pathways involving structural and individual factors that shape persisting socioeconomic inequalities in health behavior. Accounting for temporality in the mediation analysis using data collected at three time points and including prior levels of health behavior were also main strengths of this study. Controlling for baseline health behavior accounted for mediator-outcome bias due to baseline health behavior itself along with bias due to other factors, such as baseline chronic health conditions, that are expected to influence the mediators and outcomes through baseline health behavior. We are not aware of any other studies that controlled for baseline outcomes when estimating socioeconomic inequalities in health behavior. That said, existing research, including another study using GLOBE data, has brought to light the importance of considering multiple measurements of the same health behaviors over time to explain inequalities in mortality [5, 52, 53].

The approach taken in this study likely accounted for the main sources of confounding, increasing the plausibility of the results. An implication of controlling for baseline health behavior is that the results represent the estimated changes in health behavior due to the hypothesized pathways over a 10-year period of time, from the baseline measure of health behavior in 2004 to the measure of health behavior in 2014. Given the available GLOBE data, controlling for baseline health behavior was the least biased approach to investigating whether the hypothesized orderings of determinants of health behaviors were observed in this sample. Nevertheless, two other model specifications that were explored are worth noting. Running the sequential mediation models without control for baseline health behavior demonstrated that including baseline health behaviors dampened both direct and indirect effects of educational level on health behaviors, elucidating the importance of baseline health behavior as a confounder (see Additional file 5). In addition to controlling for outcomes at baseline, controlling for mediators at baseline is recommended to help account for confounding in the estimated relationships [43, 44]. In this analysis, controlling for childhood material conditions or childhood time orientation in the analyses may have accounted for potential confounding, but these data were not available. Material conditions and time orientation at baseline would not plausibly impact educational level at baseline since education was completed by 2004. Running the sequential mediation models with additional control for baseline material conditions (time orientation was not collected in 2004) in the equation predicting material conditions as a sensitivity analysis produced similar results to the models presented in the main text (see Additional file 6). The results from these alternative sets of mediation models help illustrate that the models controlling for health behavior at baseline were best able to account for confounding and provided the least biased estimates of the relationships investigated in this study.

Efforts to limit other sources of bias in the results were imperfect. The temporal ordering between educational level, the mediators, and the outcomes was unambiguous, but both mediators were measured in 2011. Time orientation was only collected in the GLOBE study in 1991 and 2011, and the potential bias introduced by using data from the same year was considered favorable to the potential bias introduced by a gap of 20 years between measurements of the two mediators. The time gaps between the observation of educational level (2004), the mediators (2011), and the outcomes (2014) introduced the possibility that short-term effects of educational level on the mediators or of the mediators on the outcomes were not captured by the analyses. These time gaps are less problematic for time orientation, a construct that deals with the longer term, but the measures of material conditions could be associated with educational level in the short term and have a short-term influence on health behavior. The sensitivity analysis controlling for baseline material conditions (see Additional file 6) showed that accounting for the short-term influence of educational level on material conditions did not alter its association with material conditions 7 years later (in 2011). While this study may not have captured the influence of material conditions on the outcomes in the very short term, the fact that mediating effects were observed after the three-year gap between the measurement of material conditions and the outcomes strengthens the plausibility of the investigated pathways [54]. For these reasons, the bias introduced by the time gaps between educational level, the mediators, and the outcomes is expected to be limited and to be outweighed by the ability to draw conclusions about the investigated pathways based on correct temporal ordering of the variables. A downside of running the analyses using SEMs was the inability to account for potential interactions between the exposure and the mediators in their influence on the outcome. Causal mediation is able to address this issue, however, the application of these techniques to sequential mediation analyses with ordinal mediators and outcomes remains arduous. It is also possible that baseline health behavior, included as a mediator-outcome confounder, was caused by educational level, the exposure, which would violate an assumption required for causal interpretation of the sequential mediation models [42]. To our knowledge, solutions to this issue [55, 56] cannot be applied to the analyses presented in this study for two reasons: our interest in the effects of both mediators and the ordinal mediators and outcomes. Similarly, methods to address the potential bias introduced by unobserved confounders have not yet been developed for situations with ordinal outcomes and causally related mediators [57].

Two other limitations of the study concern the generalizability of the results and the validity of the measures. The GLOBE sample was representative of the population residing in the Eindhoven area, but the findings of this study may not be applicable to other populations. The self-reported measures used in the analysis introduced the possibility of measurement error, although this is expected to have been more of an issue for objective constructs (i.e. income, educational level) than for the individual subjective constructs included in this study (i.e. financial strain, self-assessed health) and to have had minimal impact on the plausibility of the results [58].

### Implications for future research and policy

While the findings should be interpreted with caution, this study found one hypothesized pathway to be more likely than the other. Future research aimed at more rigorous, causal testing of whether time orientation and material conditions mediate the relationship between SEP and health behavior is warranted. Studies investigating changes in health behavior during young adulthood, a period not captured in this study, may also be of importance. Future research that includes multiple measurements of time orientation and material conditions over time could also consider the possibility of a feedback loop between the two factors in their influence on socioeconomic inequalities in health behaviors. Another possible pathway to explore is whether and how time orientation and material conditions simultaneously interact with each other in their influence on these inequalities using data powered for including interaction terms between mediators.

The importance of the interplay between structural and individual factors in determining and reducing socioeconomic inequalities in health behaviors was supported by this study. Time orientation and material conditions have previously been shown, individually, to influence socioeconomic inequalities in health behaviors, and this study established that the process through which time orientation impacts material conditions also plays an important role in explaining these inequalities for certain health behavior-related outcomes. Estimating changes due to specific factors within a given period of time, the approach taken in this study, provides an opportunity to identify potential interventions on these factors with additional consideration for when and in what order to intervene. Rather than intervening on single factors or on multiple factors independently, policymakers seeking to reduce socioeconomic inequalities in health behaviors could be more effective by considering how multiple factors impact each other. While intervening on time orientation on a broad scale in adults may be challenging, the results from this study suggest that accounting for time orientation when designing interventions aimed at material conditions could help them lead to a larger reduction in socioeconomic inequalities in certain health behaviors. For example, offering subsidies for a specific purpose that improve the ability to cover the costs of basic necessities, such as housing subsidies, may be effective in enabling healthy behavior for individuals regardless of their time orientation. More general interventions aimed at material conditions, such as direct payments, however, may be less effective for those with a present time orientation, as these could be used for other purposes with more immediate benefits.

## Conclusions

This study found evidence of a pathway from educational level to health behavior through time orientation followed by material conditions for smoking, sports participation, and self-assessed health. A study design that accounted for temporality and mediator-outcome confounding highlighted the need for future research on the determinants of socioeconomic inequalities in health behaviors to leverage longitudinal data in order to draw less biased conclusions. Based on the results from this study, policies aimed at improving socioeconomic inequalities in health behavior may be more effective when they account for the interplay between structural and individual factors and consider that certain health behaviors may be more malleable than others during adulthood.

## Supplementary Information

**Additional file 1.** Details about the GLOBE sample selection. In this additional file, the GLOBE sampling strategy and selection are described in detail.

**Additional file 2.** Overview of variables included in the analyses. A table containing all variables used in the analyses, including the description, year, survey question, and possible answer choices is presented.

**Additional file 3.** Full overview of direct and indirect effects estimated in the mediation models. Results including the estimated indirect effects through the sequence of mediators, total indirect effects, direct effects, and total effects for each of the two hypotheses investigated in the study are presented in this additional file.

**Additional file 4.** Handling of missing data. This additional file presents a detailed description of the approach to handling missing data, including a justification of the approach.

**Additional file 5.** Mediation models without control for baseline health behavior. Results of mediation models without control for baseline health behavior are presented, and differences in results from the models presented in the main text are described and interpreted.

**Additional file 6.** Mediation models with additional control for baseline material conditions. Results of mediation models with additional control for baseline material conditions are presented, the rationale for running these models is elaborated upon, and differences in results from the models presented in the main text are described and interpreted.

## Data Availability

The dataset generated and/or analysed during the current study is not publicly available due privacy regulations but is available from the corresponding author on reasonable request.

## Abbreviations

BMI: Body mass index
GLOBE: Dutch acronym for Health and Living Conditions of the Population of Eindhoven and surroundings
ISCED: International Standard Classification of Education
SEM: Structural equation model
SEP: Socioeconomic position
SQUASH: Short QUestionnaire to ASsess Health-enhancing physical activity
