# Supplementary material for: Socioeconomic inequalities in health behaviors: exploring mediation pathways through material conditions and time orientation
Source: Int J Equity Health. 2021 Aug 14;20:184. doi: 10.1186/s12939-021-01522-2 (PMC8364086; doi:10.1186/s12939-021-01522-2)
Supplement: Supplementary file 1 — Additional file 1. Details about the GLOBE sample selection. In this additional file, the GLOBE sampling strategy and selection are described in detail. [file 12939_2021_1522_MOESM1_ESM.docx]

# Additional File 1: Details about the GLOBE sample selection

The GLOBE (Dutch acronym for “Health and Living Conditions of the Population of Eindhoven and surroundings”) study is a prospective cohort study focused on understanding socioeconomic inequalities in a representative sample of the population in the Eindhoven area of the Netherlands. Postal surveys or interviews were collected in 1991, 1997, 2004, 2011, and 2014, with another wave of data collection planned for late 2021.

At baseline (1991), a postal questionnaire was sent to a random sample of non-institutionalized Dutch persons aged 14-75 years living in Eindhoven and surrounding municipalities (response rate=70.1%, N=18,973). From this sample, two subsamples were invited for subsequent stages of data collection. One subsample was chosen randomly from the baseline survey participants (response rate=79.3%, N=2,800), and the other subsample included an overrepresentation of chronically ill persons (response rate=72.3%, N=2,867). Together, these two subsamples formed the longitudinal GLOBE cohort (N=5,667), which was followed up with postal surveys in 1997, 2004, 2011 and 2014.

Along with following up the longitudinal GLOBE cohort, two additional samples were invited to participate in the 2004 GLOBE study. First, as attrition after 13 years of follow-up had become selective, a new sample (N=3,734) drawn from the same source population as the baseline sample was invited to participate in the study. Second, a sample of baseline GLOBE participants who resided in the city of Eindhoven in 1991 and still resided there in 2004 (N=2,190) was invited to fill in the 2004 postal questionnaire. The exclusion of persons who died or who had incorrect addresses (N=373) left a total of 9,898 persons who were invited to participate in 2004. With a response rate of 64.4%, data was collected for 6,377 respondents.

Between 2004 and 2011, a substantial number of participants died (N=531), emigrated from the Netherlands (N=89), or could not be traced (N=1). In 2011, all remaining respondents to the 2004 questionnaire (N=5,755) were invited to participate in the 2011 survey. The response rate was 67.1%, so data was collected from 3,863 participants.

For the 2014 postal survey, all participants in the 2004 wave who were still alive and had not emigrated were invited to participate (N=4,886). Similar to 2004, a new sample of participants, aged 25-75 years and drawn from the municipal register of the city of Eindhoven, was invited to participate in the 2014 study (N=5,782). In total, 4,851 persons responded to the 2014 postal survey (response rate=45.5%).

Data from respondents who participated in the 2004, 2011, and 2014 surveys who were at least 25 years old in 2004 (N=2,692) were used for the analyses described in this paper. More in-depth discussions of the GLOBE study design and sampling methods have been published elsewhere (1,2).

# REFERENCES

1. van Lenthe FJ, Kamphuis CBM, Beenackers MA, Jansen T, Looman CWN, Nusselder WJ, et al. Cohort Profile: Understanding socioeconomic inequalities in health and health behaviours: The GLOBE study. International Journal of Epidemiology. 2013;1–10.

2. Mackenbach JP, van de Mheen H, Stronks K. A prospective cohort study investigating the explanation of socio-economic inequalities in health in The Netherlands. Social Science & Medicine. 1994;38(2):299–308.
