# Supplementary material for: Socioeconomic inequalities in health behaviors: exploring mediation pathways through material conditions and time orientation
Source: Int J Equity Health. 2021 Aug 14;20:184. doi: 10.1186/s12939-021-01522-2 (PMC8364086; doi:10.1186/s12939-021-01522-2)
Supplement: Supplementary file 2 — Additional file 2. Overview of variables included in the analyses. A table containing all variables used in the analyses, including the description, year, survey question, and possible answer choices is presented. [file 12939_2021_1522_MOESM2_ESM.docx]

# Additional file 2: Overview of variables included in the analyses

Table 1: Overview of variables included in the analyses

| **Variable** | **Year(s)** | **GLOBE survey question(s)** | **Answer choices** |
| --- | --- | --- | --- |
|  |  |  |  |
| **Educational level** | 2004 | What is the highest grade or level of school you have completed or the highest degree you have received? | No education |
|  |  |  | Primary education |
|  |  |  | Lower vocational education |
|  |  |  | Intermediate general secondary education |
|  |  |  | Intermediate vocational education |
|  |  |  | Higher general secondary education |
|  |  |  | Higher vocational education / college |
|  |  |  | University education |
|  |  |  | Other |
|  |  |  |  |
| **Age** | 2004 | What is your age? | Open numeric answer |
|  |  |  |  |
| **Gender** | 2004 | What is your gender? | Male |
|  |  |  | Female |
|  |  |  |  |
| **Time orientation** | 2011 | Statements about the present and the future: | For each statement: |
|  |  | My day-to-day life is too busy to think about the future. | Strongly disagree |
|  |  | If I want something now, I always buy it no matter what the price. | Disagree |
|  |  | There’s no sense in thinking about the future before it gets here. | Neither agree nor disagree |
|  |  | What happens to me in the future is out of my control. | Agree |
|  |  | As long as I feel good now, I don’t worry about having health problems later in life. | Strongly agree |
|  |  | I have a plan for what I want to do in the next 5 years of my life. |  |
|  |  | I often save money to buy things I can’t afford right now. |  |
|  |  | The choices I have made in life clearly show that I think about the future. |  |
|  |  | When I plan a party or get-together, I always start weeks ahead of time. |  |
|  |  | I often think about how my actions today will affect my health when I am older. |  |
|  |  |  |  |
| **Financial strain** | 2011 | In the past year, did you have any difficulties in paying bills for food, rent, mortgage, electricity and so forth from your household income? | No, no difficulty at all |
|  |  |  | Some difficulty |
|  |  |  | Great difficulty |
|  |  |  |  |
| **Housing tenure** | 2011 | Do you live in your own home or a rented home? | Own home |
|  |  |  | Rented home, sublease |
|  |  |  |  |
| **Monthly income group in Euros** | 2011 | Please indicate how high your net household income is. | About 0 - 1200 euros a month |
|  |  |  | About 1200 - 1800 euros a month |
|  |  |  | About 1800 - 2600 euros a month |
|  |  |  | About 2600 - 4000 euros a month |
|  |  |  | More than 4000 euros a month |
|  |  |  | Unknown/ refuse to say |
|  |  |  |  |
| **Smoking status** | 2004, 2014 | Do you smoke? | Yes, I smoke … cigarettes/shags a day |
|  |  |  | Yes, I smoke … smoke pipes/cigars a day |
|  |  |  | Yes, I smoke an electronic cigarette … times a day (2014 only) |
|  |  |  | Yes, though not daily. I smoke … cigarettes/cigars a week |
|  |  |  | No, but I have smoked daily in the past |
|  |  |  | No, but I have smoked in the past every now and then (2014 only) |
|  |  |  | No, I have never smoked daily |
|  |  |  |  |
| **Sports participation** | 2004, 2014 | For each of 4 sports: |  |
|  |  | How many days in an average week in the past months did you do this sport? | Open numeric answer |
|  |  | How much time (on average) did you do this sport on a day that you did this? (hours) | Open numeric answer |
|  |  | How much time (on average) did you do this sport on a day that you did this? (minutes) | Open numeric answer |
|  |  |  |  |
| **BMI** | 2004, 2014 | What is your weight? (kilograms) | Open numeric answer |
|  |  | What is your height? (centimeters) | Open numeric answer |
|  |  |  |  |
| **Self-assessed health** | 2004, 2014 | In general, would you say your health is | Excellent |
|  |  |  | Very good |
|  |  |  | Good |
|  |  |  | Fair |
|  |  |  | Poor |

BMI: body mass index
