# Supplementary material for: Socioeconomic inequalities in health behaviors: exploring mediation pathways through material conditions and time orientation
Source: Int J Equity Health. 2021 Aug 14;20:184. doi: 10.1186/s12939-021-01522-2 (PMC8364086; doi:10.1186/s12939-021-01522-2)
Supplement: Supplementary file 3 — Additional file 3. Full overview of direct and indirect effects estimated in the mediation models. Results including the estimated indirect effects through the sequence of mediators, total indirect effects, direct effects, and total effects for each of the two hypotheses investigated in the study are presented in this additional file. [file 12939_2021_1522_MOESM3_ESM.docx]

# Additional File 3: Full overview of direct and indirect effects estimated in the mediation models

Table 1: Mediation results, Hypothesis 1: Educational level → Material conditions → Time orientation → Health behavior

| **Outcomes (in separate**  **models)** |  |  | **Indirect effects** | | | | **Direct effect** | **Total effect** | **Model fit statistics** | |
| --- | --- | --- | --- | --- | --- | --- | --- | --- | --- | --- |
|  | *N* |  | IE(M1-M2) | IE (M1) | IE(M2) | TIE | DE | TE | Chi-square | RMSEA |
| *Health behaviors* |  |  |  |  |  |  |  |  |  |  |
| **Smoking** | *2,661* |  |  |  |  |  |  |  |  |  |
| Financial strain → Time orientation |  |  | -0.001 | -0.059*** | 0.008 | -0.052** | -0.010 | -0.062 | 0.000 | 0.058 |
| Housing tenure → Time orientation |  |  | 0.001 | -0.082*** | 0.010 | -0.071** | 0.009 | -0.062 | 0.000 | 0.057 |
| Income → Time orientation |  |  | 0.003 | -0.106*** | 0.012 | -0.091*** | 0.009 | -0.082 | 0.000 | 0.074 |
| **Sports participation** | *2,420* |  |  |  |  |  |  |  |  |  |
| Financial strain → Time orientation |  |  | -0.001 | 0.039*** | 0.012* | 0.050*** | 0.064** | 0.114*** | 0.000 | 0.088 |
| Housing tenure → Time orientation |  |  | 0.001 | 0.044** | 0.007 | 0.053*** | 0.062* | 0.114*** | 0.000 | 0.086 |
| Income → Time orientation |  |  | 0.002 | 0.062*** | 0.006 | 0.069*** | 0.043 | 0.112*** | 0.000 | 0.090 |
| *Health behavior-related outcomes* | | | |  |  |  |  |  |  |  |
| **BMI** | *2,630* |  |  |  |  |  |  |  |  |  |
| Financial strain → Time orientation |  |  | 0.000 | -0.012 | -0.002 | -0.013 | -0.058* | -0.072*** | 0.001 | 0.050 |
| Housing tenure → Time orientation |  |  | 0.000 | -0.008 | -0.001 | -0.009 | -0.063** | -0.072*** | 0.000 | 0.052 |
| Income → Time orientation |  |  | 0.000 | -0.019 | 0.001 | -0.019 | -0.046 | -0.065** | 0.306 | 0.009 |
| **Self-assessed health** | *2,674* |  |  |  |  |  |  |  |  |  |
| Financial strain → Time orientation |  |  | 0.001 | 0.032*** | -0.009* | 0.024** | 0.145*** | 0.169*** | 0.000 | 0.099 |
| Housing tenure → Time orientation |  |  | -0.001 | 0.057*** | -0.010** | 0.046*** | 0.132*** | 0.169*** | 0.000 | 0.063 |
| Income → Time orientation |  |  | -0.002 | 0.070*** | -0.008** | 0.059*** | 0.095*** | 0.154*** | 0.000 | 0.093 |

BMI: body mass index, DE: direct effect, IE(M1-M2): indirect effect through material conditions followed by time orientation, IE (M1): indirect effect through material conditions only, IE(M2): indirect effect through time orientation only, RMSEA: Root Mean Square Error of Approximation, TE: total effect, TIE: total indirect effect (through material conditions only, through time orientation only, and through material conditions followed by time orientation).

Reported effects are statistically significant at *α=0.1, **α=0.05, ***α=0.01.

Notes 1 (Description of models): Results are shown for each of twelve separate models testing the effects of educational level on each of the four outcomes (smoking, sports participation, BMI, self-assessed health) through each of the three measures of material conditions (financial strain, housing tenure, income) followed by time orientation. In the models, all of which include the mediators, indirect effects refer to the effect of educational level on health behavior through the mediators, direct effects refer to the effect of educational level on health behavior that is not through the sequence of mediators, and total effects refer to the effect of educational level on health behavior both through and not through the mediators.

Notes 2 (Interpretation of model fit statistics): The chi-square test statistic describes the general fit of the model. All models except one have a good fit to the data (p-values < 0.01). The RMSEA is an absolute measure of fit, with lower values indicating a better fit to the data. Thresholds of 0.10 or 0.08 are often used to indicate sufficient fit to the data; RMSEA values for all models are below 0.10, and most are below 0.08. The Comparative Fit Index (CFI) is another commonly reported fit index for structural equation models, however we do not report it here because it is likely not informative for our models. Since the CFI is an incremental fit index, when the null model RMSEA is below about 0.158, the CFI should not be computed as it will likely be very low and, therefore, not informative. We calculated the null model RMSEA for each model using each of the 20 imputed datasets, then calculated a mean null model RMSEA for each model. The resulting null model RMSEAs were all well below the threshold of 0.158 (range 0.009-0.077).

Table 2: Mediation results, Hypothesis 2: Educational level → Time orientation → Material conditions → Health behavior

| **Outcomes (in separate**  **models)** |  |  | **Indirect effects** | | | | **Direct effect** | **Total effect** | **Model fit statistics** | |
| --- | --- | --- | --- | --- | --- | --- | --- | --- | --- | --- |
|  | *N* |  | IE(M1-M2) | IE(M1) | IE(M2) | TIE | DE | TE | Chi-square | RMSEA |
| *Health behaviors* |  |  |  |  |  |  |  |  |  |  |
| **Smoking** | *2,661* |  |  |  |  |  |  |  |  |  |
| Time orientation → Financial strain |  |  | 0.002 | 0.007 | -0.061*** | -0.052** | -0.010 | -0.062 | 0.000 | 0.058 |
| Time orientation → Housing tenure |  |  | -0.001 | 0.011 | -0.081*** | -0.071** | 0.009 | -0.062 | 0.000 | 0.057 |
| Time orientation → Income |  |  | -0.002** | 0.015 | -0.103*** | -0.091*** | 0.009 | -0.082 | 0.000 | 0.074 |
| **Sports participation** | *2,420* |  |  |  |  |  |  |  |  |  |
| Time orientation → Financial strain |  |  | -0.002* | 0.011* | 0.041*** | 0.050*** | 0.064** | 0.114*** | 0.000 | 0.088 |
| Time orientation → Housing tenure |  |  | 0.001 | 0.008 | 0.043** | 0.052*** | 0.062* | 0.114*** | 0.000 | 0.086 |
| Time orientation → Income |  |  | 0.001* | 0.008 | 0.060*** | 0.069*** | 0.043 | 0.112*** | 0.000 | 0.090 |
| *Health behavior-related outcomes* | | | |  |  |  |  |  |  |  |
| **BMI** | *2,630* |  |  |  |  |  |  |  |  |  |
| Time orientation → Financial strain |  |  | 0.000 | -0.001 | -0.012 | -0.013 | -0.059* | -0.072*** | 0.001 | 0.050 |
| Time orientation → Housing tenure |  |  | 0.000 | -0.001 | -0.008 | -0.009 | -0.063** | -0.072*** | 0.000 | 0.052 |
| Time orientation → Income |  |  | 0.000 | 0.001 | -0.019 | -0.019 | -0.046 | -0.065** | 0.306 | 0.009 |
| **Self-assessed health** | *2,674* |  |  |  |  |  |  |  |  |  |
| Time orientation → Financial strain |  |  | -0.001* | -0.008* | 0.033*** | 0.024** | 0.146*** | 0.169*** | 0.000 | 0.099 |
| Time orientation → Housing tenure |  |  | 0.001 | -0.011** | 0.056*** | 0.046*** | 0.123*** | 0.169*** | 0.000 | 0.063 |
| Time orientation → Income |  |  | 0.002** | -0.010** | 0.067*** | 0.059*** | 0.095*** | 0.154*** | 0.000 | 0.093 |

BMI: body mass index, DE: direct effect, IE(M1-M2): indirect effect through time orientation followed by material conditions, IE(M1): indirect effect through time orientation only, IE(M2): indirect effect through material conditions only, RMSEA: Root Mean Square Error of Approximation, TE: total effect, TIE: total indirect effect (through time orientation, through material conditions, and through time orientation followed by material conditions).

Reported effects are statistically significant at *α=0.1, **α=0.05, ***α=0.01.

Notes 1 (Description of models): Results are shown for each of twelve separate models testing the effects of educational level on each of the four outcomes (smoking, sports participation, BMI, self-assessed health) through time orientation followed by each of the three measures of material conditions (financial strain, housing tenure, income). In the models, all of which include the mediators, indirect effects refer to the effect of educational level on health behavior through the mediators, direct effects refer to the effect of educational level on health behavior that is not through the sequence of mediators, and total effects refer to the effect of educational level on health behavior both through and not through the mediators.

Notes 2 (Interpretation of model fit statistics): The chi-square test statistic describes the general fit of the model. All models except one have a good fit to the data (p-values < 0.01). The RMSEA is an absolute measure of fit, with lower values indicating a better fit to the data. Thresholds of 0.10 or 0.08 are often used to indicate sufficient fit to the data; RMSEA values for all models are below 0.10, and most are below 0.08. The Comparative Fit Index (CFI) is another commonly reported fit index for structural equation models, however we do not report it here because it is likely not informative for our models. Since the CFI is an incremental fit index, when the null model RMSEA is below about 0.158, the CFI should not be computed as it will likely be very low and, therefore, not informative. We calculated the null model RMSEA for each model using each of the 20 imputed datasets, then calculated a mean null model RMSEA for each model. The resulting null model RMSEAs were all well below the threshold of 0.158 (range 0.009-0.077).
