# Supplementary material for: Socioeconomic inequalities in health behaviors: exploring mediation pathways through material conditions and time orientation
Source: Int J Equity Health. 2021 Aug 14;20:184. doi: 10.1186/s12939-021-01522-2 (PMC8364086; doi:10.1186/s12939-021-01522-2)
Supplement: Supplementary file 4 — Additional file 4. Handling of missing data. This additional file presents a detailed description of the approach to handling missing data, including a justification of the approach. [file 12939_2021_1522_MOESM4_ESM.docx]

# Additional File 4: Handling of missing data

A total of 3.3% of the values from all variables used in the analyses were missing. Sports participation in 2004 and 2014 and time orientation in 2011 had 7.1%, 10.1%, and 20.2% missing values, respectively. Bias introduced into the analyses by missing data was accounted for using multiple imputation.

All variables were imputed, then respondents with missing data for a given outcome were excluded from the mediation analyses predicting that outcome. This approach ensured that all available information was used during multiple imputation and that an efficient variance system was generated. Since the purpose of multiple imputation is to generate data, which is separate from the analyses the data will be used for, it was important to include information on other variables from respondents with missing values for the analysis outcome variables. Since the imputed outcome values were determined (in part) by the exposure, mediators, and confounders, the mediation analyses for a given outcome excluded respondents with missing values in the original data for that outcome.

Key dependent variables in the analyses had relatively high percentages of missing data and the aim of the analyses was to draw conclusions about the distribution of the estimated parameters, so 20 imputed datasets were generated using an efficient predictor set (average of 20.78 predictors) (1). The sequential mediation SEMs were estimated using each of the 20 imputed datasets, then parameter estimates and standard errors were pooled using Rubin’s rule (2).

# REFERENCES

1. van Buuren S. Flexible Imputation of Missing Data. 2nd ed. Chapman & Hall/CRC; 2018.

2. Rubin D. Multiple Imputation for Nonresponse in Surveys. New York: John Wiley & Sons; 1987.
