# Supplementary material for: Socioeconomic inequalities in health behaviors: exploring mediation pathways through material conditions and time orientation
Source: Int J Equity Health. 2021 Aug 14;20:184. doi: 10.1186/s12939-021-01522-2 (PMC8364086; doi:10.1186/s12939-021-01522-2)
Supplement: Supplementary file 5 — Additional file 5. Mediation models without control for baseline health behavior. Results of mediation models without control for baseline health behavior are presented, and differences in results from the models presented in the main text are described and interpreted. [file 12939_2021_1522_MOESM5_ESM.docx]

# Additional File 5: Mediation models without control for baseline health behavior

The results from the models without control for baseline health behavior (see Table 1 and Table 2 below) are markedly different from those with control for baseline health behavior, which are presented in the main text. The direct effect sizes are substantially larger in the models without control for baseline health behavior, especially for smoking and BMI. The direct effects of educational level on smoking are highly statistically significant for the models including financial strain or housing tenure, while no significant direct effects were observed in the models controlling for baseline smoking. These findings imply that a substantial part of the effects of educational level on health behaviors (especially smoking and BMI) observed in the models presented in this additional file were in fact due to baseline health behavior or underlying factors captured by baseline health behavior, not educational level.

In models both with and without control for baseline health behavior, there is little evidence of a pathway from educational level to health behavior through material conditions followed by time orientation. In the models presented here, income followed by time orientation appears to play a weakly significant role in the relationship between educational level and self-assessed health (see Table 1). Compared to the models controlling for baseline health behavior, the models without additional control show stronger evidence of a pathway from educational level to health behavior through time orientation followed by material conditions, both in terms of stronger statistical significance and a larger number of significant indirect effects (see Table 2). In these models without control for baseline health behavior, indirect effects for smoking (for financial strain and income), BMI (for housing), sports participation (all measures of material conditions), and self-assessed health (for financial strain and income) were statistically significant.

The differences in findings between models with and without control for baseline health behavior highlight that baseline health behavior is an important confounder of the relationships examined in this study. The results presented in this additional file are less plausible than those presented in the main text because omitting control for baseline health behavior prevents any insight into changes in health behavior over time and overestimates the effects of educational level on health behavior through the mediators.

Table 1: Mediation results, Hypothesis 1: Educational level → Material conditions → Time orientation → Health behavior

| **Outcomes (in separate**  **models)** |  |  | **Measure of material conditions (tested in separate models)** | | | | | |
| --- | --- | --- | --- | --- | --- | --- | --- | --- |
|  |  |  | **Financial strain → Time orientation** | | **Housing tenure → Time orientation** | | **Income group → Time orientation** | |
|  | *N* |  | IE | DE | IE | DE | IE | DE |
| *Health behaviors* |  |  |  |  |  |  |  |  |
| **Smoking** | *2,661* |  | 0.000 | -0.116*** | 0.000 | -0.092** | 0.001 | -0.059 |
| **Sports participation** | *2,420* |  | -0.001 | 0.097*** | 0.001 | 0.085*** | 0.002 | 0.066** |
| *Health behavior-related outcomes* | | | |  |  |  |  |  |
| **BMI** | *2,630* |  | 0.000 | -0.140*** | 0.000 | -0.131*** | 0.000 | -0.146*** |
| **Self-assessed health** | *2,674* |  | 0.001 | 0.193*** | -0.001 | 0.178*** | -0.002* | 0.143*** |

BMI: body mass index, DE: direct effect, IE: indirect effect through material conditions followed by time orientation.

Reported effects are statistically significant at *α=0.1, **α=0.05, ***α=0.01.

Notes: Results are shown for each of twelve separate models testing the effects of educational level on each of the four outcomes (smoking, sports participation, BMI, self-assessed health) through each of the three measures of material conditions (financial strain, housing tenure, income) followed by time orientation. In the models, all of which include the mediators, direct effects refer to the effect of educational level on health behavior that is not through the sequence of mediators and indirect effects refer to the effect of educational level on health behavior through the sequence of mediators.

Table 2: Mediation results, Hypothesis 2: Educational level → Time orientation → Material conditions → Health behavior

| **Outcomes (in separate**  **models)** |  |  | **Measure of material conditions (tested in separate models)** | | | | | |
| --- | --- | --- | --- | --- | --- | --- | --- | --- |
|  |  |  | **Time orientation → Financial strain** | | **Time orientation → Housing tenure** | | **Time orientation → Income group** | |
|  | *N* |  | IE | DE | IE | DE | IE | DE |
| *Health behaviors* |  |  |  |  |  |  |  |  |
| **Smoking** | *2,661* |  | 0.003* | -0.116*** | -0.002 | -0.092** | -0.003** | -0.059 |
| **Sports participation** | *2,420* |  | -0.002** | 0.097*** | 0.002* | 0.085*** | 0.002** | 0.067** |
| *Health behavior-related outcomes* | | | |  |  |  |  |  |
| **BMI** | *2,630* |  | 0.001* | -0.140*** | -0.001 | -0.131*** | -0.001 | -0.146*** |
| **Self-assessed health** | *2,674* |  | -0.002** | 0.193*** | 0.002 | 0.178*** | 0.002** | 0.143*** |

BMI: body mass index, DE: direct effect, IE: indirect effect through time orientation followed by material conditions.

Reported effects are statistically significant at *α=0.1, **α=0.05, ***α=0.01.

Notes: Results are shown for each of twelve separate models testing the effects of educational level on each of the four outcomes (smoking, sports participation, BMI, self-assessed health) through time orientation followed by each of the three measures of material conditions (financial strain, housing tenure, income). In the models, all of which include the mediators, direct effects refer to the effect of educational level on health behavior that is not through the sequence of mediators and indirect effects refer to the effect of educational level on health behavior through the sequence of mediators.
