# Supplementary material for: Socioeconomic inequalities in health behaviors: exploring mediation pathways through material conditions and time orientation
Source: Int J Equity Health. 2021 Aug 14;20:184. doi: 10.1186/s12939-021-01522-2 (PMC8364086; doi:10.1186/s12939-021-01522-2)
Supplement: Supplementary file 6 — Additional file 6. Mediation models with additional control for baseline material conditions. Results of mediation models with additional control for baseline material conditions are presented, the rationale for running these models is elaborated upon, and differences in results from the models presented in the main text are described and interpreted. [file 12939_2021_1522_MOESM6_ESM.docx]

# Additional File 6: Mediation models with additional control for baseline material conditions

Material conditions at baseline (2004) were not considered plausible exposure-mediator confounders in the first hypothesized pathway. Baseline material conditions would be unlikely to confound the relationship between educational level (2004) and material conditions (2011) since education was completed before 2004. However, it is possible that baseline material conditions are a common cause of time orientation (2011) and material conditions (2011), acting as a mediator-mediator confounder in the second hypothesized pathway. However, it was not possible to include baseline time orientation as a mediator-mediator confounder in the models testing the first hypothesized pathway to achieve consistency between the models testing the two hypotheses. For these reasons, baseline material conditions were not included in the models presented in the main text.

That said, adding baseline material conditions to the models substantially improved the models’ fit to the data, especially those predicting sports participation and self-assessed health. Results from these models were very similar to those described in the main text except for the indirect effect of educational level on sports participation through time orientation followed by income, which was no longer significant (see Table 1 and Table 2 below). Since baseline material conditions were not considered conceptually sound confounders, the results from the models controlling for baseline age, gender, and health behavior were interpreted as the most plausible and reliable.

Table 1: Mediation results, Hypothesis 1: Educational level → Material conditions → Time orientation → Health behavior

| **Outcomes (in separate**  **models)** |  |  | **Measure of material conditions (tested in separate models)** | | | | | |
| --- | --- | --- | --- | --- | --- | --- | --- | --- |
|  |  |  | **Financial strain → Time orientation** | | **Housing tenure → Time orientation** | | **Income group → Time orientation** | |
|  | *N* |  | IE | DE | IE | DE | IE | DE |
| *Health behaviors* |  |  |  |  |  |  |  |  |
| **Smoking** | *2,661* |  | 0.000 | -0.014 | 0.000 | -0.029 | 0.001 | -0.024 |
| **Sports participation** | *2,420* |  | -0.001 | 0.062* | 0.000 | 0.065** | 0.001 | 0.035 |
| *Health behavior-related outcomes* | | | |  |  |  |  |  |
| **BMI** | *2,630* |  | 0.000 | -0.056* | 0.000 | -0.073** | 0.000 | -0.057 |
| **Self-assessed health** | *2,674* |  | 0.000 | 0.142*** | 0.000 | 0.143*** | -0.001 | 0.100*** |

BMI: body mass index, DE: direct effect, IE: indirect effect through material conditions followed by time orientation.

Reported effects are statistically significant at *α=0.1, **α=0.05, ***α=0.01.

Notes: Results are shown for each of twelve separate models testing the effects of educational level on each of the four outcomes (smoking, sports participation, BMI, self-assessed health) through each of the three measures of material conditions (financial strain, housing tenure, income) followed by time orientation. In the models, all of which include the mediators, direct effects refer to the effect of educational level on health behavior that is not through the sequence of mediators and indirect effects refer to the effect of educational level on health behavior through the sequence of mediators.

Table 2: Mediation results, Hypothesis 2: Educational level → Time orientation → Material conditions → Health behavior

| **Outcomes (in separate**  **models)** |  |  | **Measure of material conditions (tested in separate models)** | | | | | |
| --- | --- | --- | --- | --- | --- | --- | --- | --- |
|  |  |  | **Time orientation** → **Financial strain** | | **Time orientation** → **Housing tenure** | | **Time orientation** → **Income group** | |
|  | *N* |  | IE | DE | IE | DE | IE | DE |
| *Health behaviors* |  |  |  |  |  |  |  |  |
| **Smoking** | *2,661* |  | 0.002 | -0.014 | 0.000 | -0.030 | -0.001 | -0.024 |
| **Sports participation** | *2,420* |  | -0.002* | 0.061* | 0.000 | 0.065** | 0.001 | 0.035 |
| *Health behavior-related outcomes* | | | |  |  |  |  |  |
| **BMI** | *2,630* |  | 0.000 | -0.056* | 0.000 | -0.073** | 0.000 | -0.056 |
| **Self-assessed health** | *2,674* |  | -0.001* | 0.141*** | 0.000 | 0.143*** | 0.001* | 0.100*** |

BMI: body mass index, DE: direct effect, IE: indirect effect through time orientation followed by material conditions.

Reported effects are statistically significant at *α=0.1, **α=0.05, ***α=0.01.

Notes: Results are shown for each of twelve separate models testing the effects of educational level on each of the four outcomes (smoking, sports participation, BMI, self-assessed health) through time orientation followed by each of the three measures of material conditions (financial strain, housing tenure, income). In the models, all of which include the mediators, direct effects refer to the effect of educational level on health behavior that is not through the sequence of mediators and indirect effects refer to the effect of educational level on health behavior through the sequence of mediators.
